# What lies behind the large genome of *Colletotrichum lindemuthianum*

**DOI:** 10.3389/ffunb.2024.1459229

**Published:** 2024-10-15

**Authors:** Leandro Lopes da Silva, Hilberty Lucas Nunes Correia, Osiel Silva Gonçalves, Pedro Marcus Pereira Vidigal, Rafael Oliveira Rosa, Mateus Ferreira Santana, Marisa Vieira de Queiroz

**Affiliations:** ^1^ LGMM, Departamento de Microbiologia, Instituto de Biotecnologia Aplicada à Agropecuária (BIOAGRO), Universidade Federal de Viçosa, Viçosa, Brazil; ^2^ Crop Production and Pest Control Research Unit, Agricultural Research Service, United States Department of Agriculture (USDA), West Lafayette, IN, United States; ^3^ NuBioMol, Centro de Ciências Biológicas, Universidade Federal de Viçosa, Viçosa, Brazil

**Keywords:** common bean anthracnose, comparative genomics, repetitive elements, repeat-induced point (RIP) mutations, long read DNA sequencing

## Abstract

*Colletotrichum lindemuthianum* is the etiological agent of anthracnose disease in common bean (*Phaseolus vulgaris* L.), noted for its ability to cause serious damage and significant pathogenic variability. This study reveals the features of the high-quality genome of *C. lindemuthianum*. Analysis showed improvements over the first assembly, with the refined genome having 119 scaffolds, ten times fewer than the first, and a 19% increase in gene number. The effector candidates increased by nearly 1.5 times. More than 40% of the amino acid sequences with homologs in the Pathogen-Host Interactions (PHI-base) database are linked to pathogenicity. Of 18 putative proteins identified as Chitinase-like Protein, six have a mutation in the enzyme catalytic motif, and three showed gene expression in the biotrophic phase, indicating they can act as effectors. Comparative genomic analyses with 30 other fungal species revealed that *C. lindemuthianum* is among the top three fungi encoding transport proteins. Seven Necrosis and Ethylene-Inducing Peptide 1 (Nep1)-Like Proteins (NLPs) are present in the *C. lindemuthianum* genome, but none had complete identity with the GHRHDWE conserved motif of NLPs; two were grouped with proteins that induce necrosis and may retain the capability to induce host necrosis. *Colletotrichum* species show a high number of secondary metabolite (SM) clusters, with *C. lindemuthianum* having 47 SM clusters. Approximately 60% of the *C. lindemuthianum* genome is composed of repetitive elements, a significantly higher proportion than in other fungi. These differences in transposable element (TE) numbers may explain why *C. lindemuthianum* has one of the largest genomes among the fungi analyzed. A significant portion of its genome comprises retroelements, particularly the *Ty1/Copia* superfamily, which accounts for 22% of the genome and represents 40% of the repetitive elements. The genomic profile features a remarkably high RIP-affected genomic proportion of 54.77%, indicating substantial RIP activity within this species. This high-quality genome of *C. lindemuthianum*, a significant pathogen in common bean cultivation, will support future research into this pathosystem, fostering a deeper understanding of the interaction between the fungus and its host.

## 1 Introduction

Fungi from the *Colletotrichum* genus are responsible for causing anthracnose in crops worldwide, being a relevant pathogen in both pre-harvest and post-harvest, mainly in tropical and subtropical regions (Cai et al., 2011). The genus is among the ten most relevant phytopathogenic fungi when considering its biological characteristics and economic impact (Dean et al., 2012). It is also recognized as a great model for studies on host-pathogen interaction (Perfect et al., 1999).

Although Brazil is among the top three bean (*Phaseolus vulgaris* L.) producers in the world (FAOSTAT, 2022), several factors can contribute to decreasing its productivity, among them, diseases caused by fungi, such as anthracnose (Perseguini et al., 2016). The common bean anthracnose caused by *Colletotrichum lindemuthianum* (Saccardo & Magnus) Briosi & Cavara is one of the major diseases of this crop in Brazil, which can cause significant losses in production (Barcelos et al., 2014). With favorable conditions of humidity and temperature, anthracnose results in premature defoliation, premature flowering, and pods falling, and it can lead to plant death in extreme cases, decreasing productivity and causing grain depreciation (Sharma et al., 2008).

The fungus *C. lindemuthianum* has high genetic variability, with at least 182 physiological races identified in different world regions (Padder et al., 2017). Thus, over the years, several studies investigated the genetic and pathogenic variability of *C. lindemuthianum* (Mahuku and Riascos, 2004; Sharma et al., 2007; Ishikawa et al., 2010; Pinto et al., 2012). This fungus and its association with common bean has been studied with research focusing on the cytology and physiology of common bean infection (O’Connell et al., 1985, 1996; O’Connell, 1987; O’Connell and Ride, 1990); conidia anastomosis and parasexuality (Roca et al., 2003; Ishikawa et al., 2010, 2013; Rosada et al., 2010); sexual reproduction (Souza et al., 2010); differential gene expression during pathogenesis (Fontenelle et al., 2017; Romero et al., 2024); identification of genes required for pathogenicity (Parisot et al., 2002; Pellier et al., 2003; Hoi et al., 2007; Cnossen-Fassoni et al., 2013; Soares et al., 2014; Nogueira et al., 2019); analysis of mtDNA and genes encoding effector proteins and pectinases (Queiroz et al., 2018, 2019; Silva et al., 2021, 2022), among others.

With the expanding knowledge of fungal genomes, comparative genomics can be applied to analyze the organization and composition of the *Colletotrichum* genome, facilitating a better understanding of its relationship to lifestyle. Several gene families are more abundant in *Colletotrichum* genomes than observed in other phytopathogenic fungi. Comparative genomics studies make it possible to analyze the gene repertoire linked to secondary metabolism, the enzymes related to plant cell wall degradation, pathogen virulence, and effector candidates to understand the pathogenic process (O’Connell et al., 2012; Gan et al., 2016; Rao and Nandineni, 2017; Bhadauria et al., 2019; Lelwala et al., 2019). In addition, *Colletotrichum* species have a high potential for secondary metabolite production, surpassing several other phytopathogenic fungi (Gan et al., 2012; O’Connell et al., 2012; Baroncelli et al., 2016; Liang et al., 2018).

The orbiculare complex has hemibiotrophic species, which cause diseases in herbaceous plants. Among these species, *Colletotrichum orbiculare*, *C. lindemuthianum*, *Colletotrichum trifolii*, *Colletotrichum spinosum*, and *Colletotrichum sidae* have already had their genomes sequenced (Queiroz et al., 2017; Gan et al., 2019). These species have distinct characteristics from other *Colletotrichum* species, such as an expansion in genome size not accompanied by gene number increase and G+C content (%) reduction (Gan et al., 2012, 2019; Queiroz et al., 2017).

Most *Colletotrichum* genomes have been sequenced using the Illumina platform, including *C. lindemuthianum* (Queiroz et al., 2017), but these genomes have gaps due to small and repetitive reads which are difficult to assemble using this technology (English et al., 2012). The *C. lindemuthianum* genomes sequenced are among the largest ever reported in the *Colletotrichum* genus (Queiroz et al., 2017), surpassed only by the *C. trifolii* genome (Gan et al., 2019), which is a drawback when trying to obtain genomes with better quality. Alternative sequencing strategies, such as long read sequencing, facilitate the assembly of larger genomes (English et al., 2012). Due to the importance of *C. lindemuthianum* as a phytopathogen, the objective of the present work was to present a high-quality genome to reveal its remarkable genomic characteristics and to identify virulence factors involved in common bean infection through comparative genomics with other fungi.

## 2 Materials and methods

### 2.1 Microorganism and growth conditions

The *C. lindemuthianum* isolate A_2_ 2-3 physiological race 89 analyzed in this study is from the collection of the Laboratory of Molecular Genetics of Microorganisms/Bioagro from Universidade Federal de Viçosa, Viçosa, Brazil. For culture maintenance and sporulation, fungal cultivation was on potato dextrose agar (PDA) (Kasvi) or YMC (malt extract 10 g.L^-1^; yeast extract 2 g.L^-1^; agar 15 g.L^-1^) medium at 22°C for six to eight days. For growth in liquid medium, approximately 10^5^ conidia.mL^-1^ were inoculated in GPYECH medium (20 g.L^-1^ glucose; 5 g.L^-1^ peptone; 1.0 g.L^-1^ yeast extract; 1.0 g.L^-1^ hydrolyzed casein) and cultured with shaking at 150-200 rpm for four days at 22°C.

### 2.2 Nucleic acid extraction and sequencing

Total DNA of the *C. lindemuthianum* isolate was extracted from fresh mycelia after four days of growth in GPYECH medium with the NucleoBond™ Buffer Set IV in combination with NucleoBond™ AXG 100 columns (Macherey-Nagel Bioanalysis™) following the manufacturer’s instructions. The *C. lindemuthianum* isolate genomic DNA was sequenced using the PacBio Sequel platform (CD Genomics, New York, United States). The RNA of bean leaves inoculated with the *C. lindemuthianum* at different times post inoculation (48 h and 120 h) and in GPYECH medium were obtained as previously described (Queiroz et al., 2019). The RNA was sequenced using the Illumina HiSeq platform with three biological replicates per time point (GenOne Soluções em Biotecnologia, Rio de Janeiro, Brazil).

### 2.3 Genome assembly

The genome assembly used a hybrid approach combining the previously short paired-read data generated using Illumina HiSeq 2500 (Queiroz et al., 2017) and long read data obtained using PacBio Sequel subreads. The quality assessment of the short reads used the reports of FastQC v. 0.11.9 (Andrews, 2010). Trimgalore v. 0.6.7 (Krueger et al., 2015) removed the library adapters using the “auto-detection” setting, and Trimmomatic v. 0.39 (Bolger et al., 2014) trimmed and filtered the reads.

The MaSuRCA v. 4.0.3 (Zimin et al., 2013) executed the hybrid assembly using trimmed short and long reads data, combining SOAPdenovo2 (Luo et al., 2012) v. 2.04 to assembly the short reads and Flye v. 2.5 (Kolmogorov et al., 2019) to assembly the long reads. The scaffolds were corrected by mapping the paired reads using the BWA-MEM algorithm of BWA v. 0.7.17 (Li and Durbin, 2009), sorting the mapping files using the Picard toolkit v. 2.26.2 (https://github.com/broadinstitute/picard), and polishing the sequences using the Pilon v. 1.24 (Walker et al., 2014), with default parameters.

### 2.4 Structural annotation

The genome annotation followed the pipeline of Funannotate v. 1.8.7 (Palmer and Stajich, 2020) and combined *ab initio* and evidence-based methods, using transcriptome data and homology with known proteins for gene prediction. First, the Repeat-Modeler v. 2.0.1 (Flynn et al., 2020) executed an *ab-initio* prediction of transposable elements (TEs) and repetitive elements to produce a library from the scaffolds. Then, RepeatMasker v. 4.1.2-p1 (Tarailo-Graovac and Chen, 2009) executed a soft-masking of these elements in the scaffold sequences using the predicted library. Before the gene prediction, the tool “train” of Funannotate aligned RNA-seq data in the scaffold sequences using the option “jaccard-clip” since high gene density in fungal genomes is expected. After training, the tool “predict” of Funnannotate predicted the genes using protein sequences of the predicted reference proteomes of the *Colletotrichum* genus (Taxon ID 5455) available in the UniProt database (https://www.uniprot.org/proteomes). Then, the tool “update” of Funnannotate added UTR data to the predictions and fixed the gene models. BUSCO (Benchmarking Universal Single-Copy Orthologs) (Simão et al., 2015) evaluated the genome and gene prediction completeness using the proteome mode and the orthologous dataset of the Fungi kingdom (fungi_odb10) and Glomerellales order (glomerellales_odb10).

### 2.5 Functional annotation

The pipeline for secretome prediction executed sequential filtering steps with the following software: SignalP v. 4.1 (Petersen et al., 2011), Target-P v. 1.1 (Emanuelsson et al., 2000), and WoLF PSORT (Horton et al., 2007) to identify signal peptides and predict the subcellular location; TMHMM v. 2.0 (Emanuelsson et al., 2007) and Phobius v. 1.1 (Käll et al., 2007) to exclude sequences with transmembrane regions; and PredGPI (Eisenhaber et al., 2004) to exclude amino acid sequences that have GPI anchors.

A search against the MEROPS database (Rawlings and Bateman, 2021) identified putative proteases and protease inhibitors, excluding sequences with incomplete domains and mutated active sites. dbCAN (Yin et al., 2012) identified the carbohydrate active enzymes (CAZymes). Within the CAZyme analysis, chitinase enzymes were presumed to belong to glycoside hydrolase family 18 (GH18). We used the first predicted proteome of *C. lindemuthianum* (Queiroz et al., 2017) and the newly predicted proteome to conduct an improved effector analysis using Predector v1.2.7 (Jones et al., 2021). The pathogenesis interactions database (PHI-base) (Winnenburg, 2006) allowed the identification of candidate pathogenicity genes, and cytochrome P450 (CYP450) enzymes were screened using the cytochrome P450 database (Nelson, 2009). A local BLAST search using the CDS and amino acid sequences of MAT1-1 from *Fusarium graminearum* (AF318048.1) and MAT1-2-1 from *C. lindemuthianum* isolate MU 03 (EU236950.1) was performed against the *C. lindemuthianum* isolate A_2_ 2-3 genome assembly to determine its mating type.

BLASTp was used to identify proteins similar to Necrosis and Ethylene-Inducing Peptide 1 (Nep1)-Like Proteins (NLPs) to identify putative homologs of known NLP proteins PsojNIP (type 1) from *Phytophthora sojae* (Qutob et al., 2002), NLPpcc (type 2) from *Pectobacterium carotovorum* subsp. *carotovorum* (Mattinen et al., 2004), and Afu5g02100 (type 3) from *Aspergillus fumigatus* (Upadhyay et al., 2012). Sequence alignments using Clustal Omega (Sievers et al., 2011) allowed the identification of the conserved consensus motif GHRHDWE in the amino acid sequence of *C. lindemuthianum* NLPs. To confirm the homology of the *C. lindemuthianum* NLP candidates, a Maximum Likelihood (ML) analysis was conducted in MEGA v. 11 (Tamura et al., 2021). The Clustal tool aligned the amino acid sequences of NLP proteins of *C. lindemuthianum* and other fungal species, and the ML analysis was performed using the JTT matrix-based substitution model with bootstraps of 10,000 replicates to infer the confidence of the phylogenetic tree.

Transporter prediction used TMHMM v. 2.0 (Emanuelsson et al., 2007) to identify proteins with transmembrane regions, and similarity searches using the BLASTp tool to search for putative homologs in the Transporter Classification Database (TCDB) (Saier et al., 2021). The Secondary Metabolite Analysis SHell (antiSMASH fungal version) (Medema et al., 2011) was used to scan the genome to identify potential secondary metabolite (SM) gene clusters, and RIPper (Van Wyk et al., 2019) was used to analyze the genome to identify Repeat-Induced Point (RIP) mutations.

To provide a broader overview of functional annotation, protein sequences annotated in genomes from 29 fungal species available in the NCBI Genome database (https://www.ncbi.nlm.nih.gov/genome/) (**Supplementary Table S1**) were used for comparative analysis. In addition, the RNA-seq data of bean leaves inoculated with the *C. lindemuthianum* isolate A_2_ 2-3 at different times post inoculation (48 h and 120 h) allowed the investigation of the expression patterns of genes involved in pathogenicity. The gene expression analysis followed the methodology previously described by Silva et al. (2022), whereas the genome of *C. lindemuthianum* was indexed using Bowtie2 v software. 2.2.8 (Langmead et al., 2009) in default settings and mapped against each transcriptome library using Tophat v.2.1.1 (Trapnell et al., 2009). Transcriptome assembly and gene expression quantification were performed using Cufflinks v.2.2.1 (Trapnell et al., 2010). The fragments per kilobase of transcript per million mapped reads (FPKM) values of the gene candidates were used to analyze gene expression levels.

## 3 Results

### 3.1 *Colletotrichum lindemuthianum* genome assembly and gene prediction

To obtain a more refined genome, a hybrid assembly was performed using the previous data generated by Illumina sequencing (Queiroz et al., 2017) and the new data from the PacBio platform. The new genome assembly has only 119 scaffolds, ten times less than the first assembly (**Table 1**). The scaffold length in the new assembly is greater than the previous one. Gene prediction was performed using Funannotate integrating specific RNA-Seq evidence from *C. lindemuthianum*, protein homology from other *Colletotrichum* species, and *ab initio* gene predictions to obtain consensus gene models. A total of 13,821 genes were predicted from the new assembly, an increment of almost 19% in the number of previously predicted genes. A total of 14,126 putative proteins were predicted from the analyzed genome. The greater putative protein number than the predicted gene number is related to alternative splicing variants. The new assembly completeness was accessed through BUSCO, which showed more than 95% completeness when compared with genes from both Fungi and Glomerellales. This new and more refined assembly makes it possible to better study and understand the *C. lindemuthianum* genome.

**Table 1:** Comparing the *C. lindemuthianum* genome assembly using different approaches.

	Illumina	Pacbio + Illumina
Scaffold number	1,276	119
N50	158 kbp	2.1 Mbp
Smallest scaffold	1.03 kbp	3.6 Kbp
Biggest scaffold	1.11 Mbp	9.3 Mbp
Genome Size	99.16 Mbp	100.48 Mbp
GC Content	37.3%	37.25%
Predicted gene number	11,627	13,821

### 3.2 Predicted secretome

The prediction of putatively secreted proteins in *C. lindemuthianum* was performed by a sequence of analyses. Initially, signal peptide prediction was performed for the predicted *C. lindemuthianum* proteins. Thus, 1,346 putative proteins showed a secretion signal and did not have the mitochondria and other sub-cellular regions predicted to be their final destination. Putative proteins that presented transmembrane helices and that had glycophosphatidylinositol anchor motifs (GPI-anchor) were also excluded. Thus, a total of 1,054 putative protein sequences were predicted to be secreted, which corresponds to approximately 7.5% of all *C. lindemuthianum* putative proteins.

### 3.3 CAZymes

**Figure 1:**
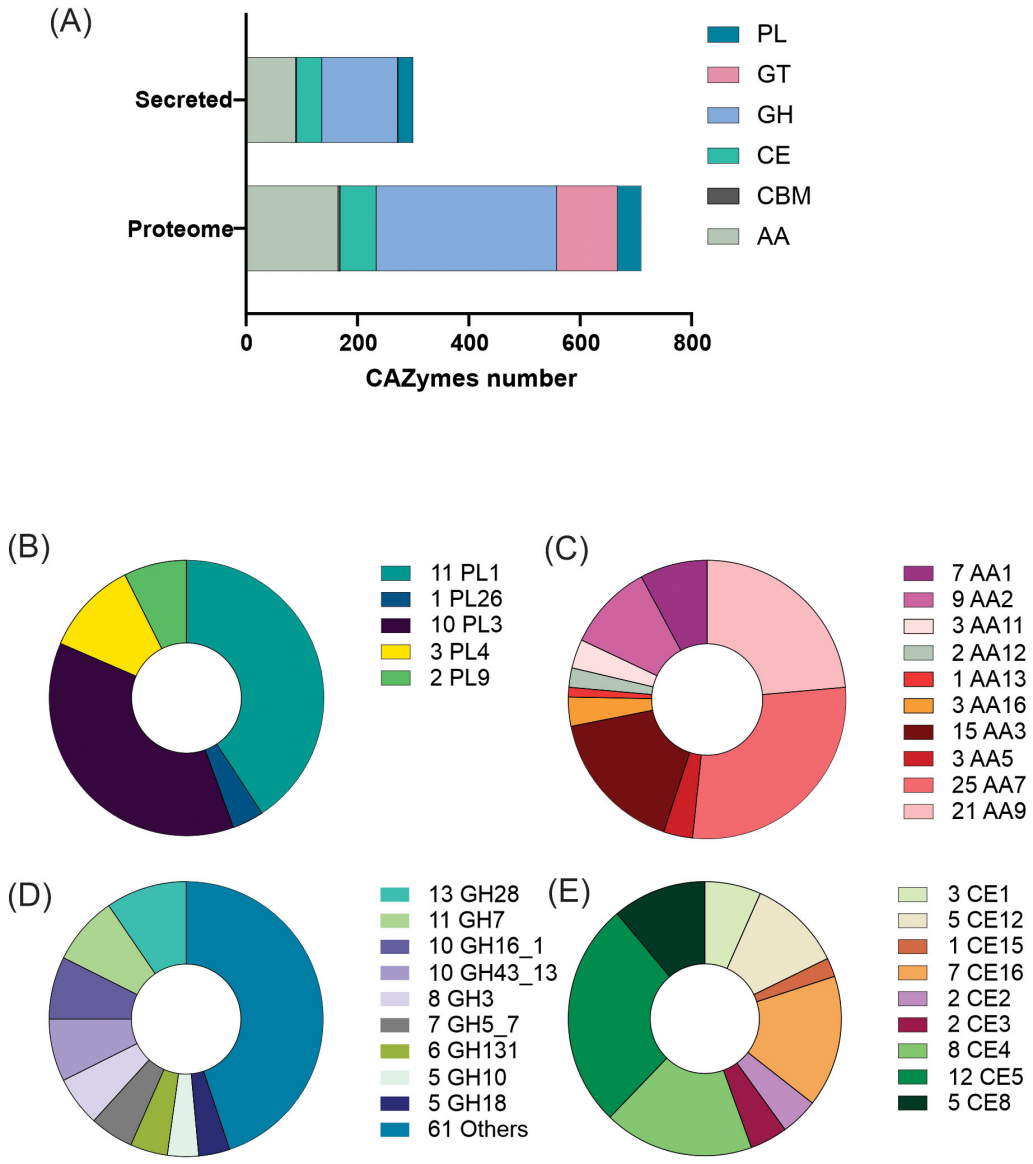
CAZymes produced by *Colletotrichum lindemuthianum*. **(A)** Total CAZymes identified in the proteome and secretome. PL, Polysaccharide Lyases; GT, Glycosyl Transferases; GH, Glycosyl Hydrolases; CBM, Carbohydrate-Binding Modules; AA, Auxiliary Activities; CE, Carbohydrate Esterases. Major families of CAZymes **(B)** Auxiliary Activities, **(C)** Polysaccharide Lyases, **(D)** Glycosyl Hydrolases and **(E)** Carbohydrate Esterases.

Seven hundred and ten putative proteins were identified as CAZymes in the *C. lindemuthianum* predicted proteome (**Figure 1**). Of these, 300 putative proteins classified into 70 CAZyme families were predicted to be secreted, corresponding to 42% of the CAZymes present in the proteome. The CAZyme classes glycosyl hydrolase (GH), polysaccharide lyase (PL), auxiliary activities (AA), and carbohydrate esterase (CE) had the greatest number of secreted protein members. The three most abundant AA families with the greatest number of members were those comprised of putative oxidases, monooxygenases, and carbohydrate dehydrogenases. The enzymes present in the three PL families with the most members had activities related to pectin degradation. The enzymes present in the four most abundant GH families with the most members had activities related to the degradation of cellulose, hemicellulose, and pectin. The enzymes present in the three CE families with the most members had activities related to xylan acetyltransferase. Of those secreted CAZymes involved in plant cell wall degradation, there was a greater abundance of enzymes related to pectin degradation than the other constituents.

### 3.4 Proteases

**Figure 2:**
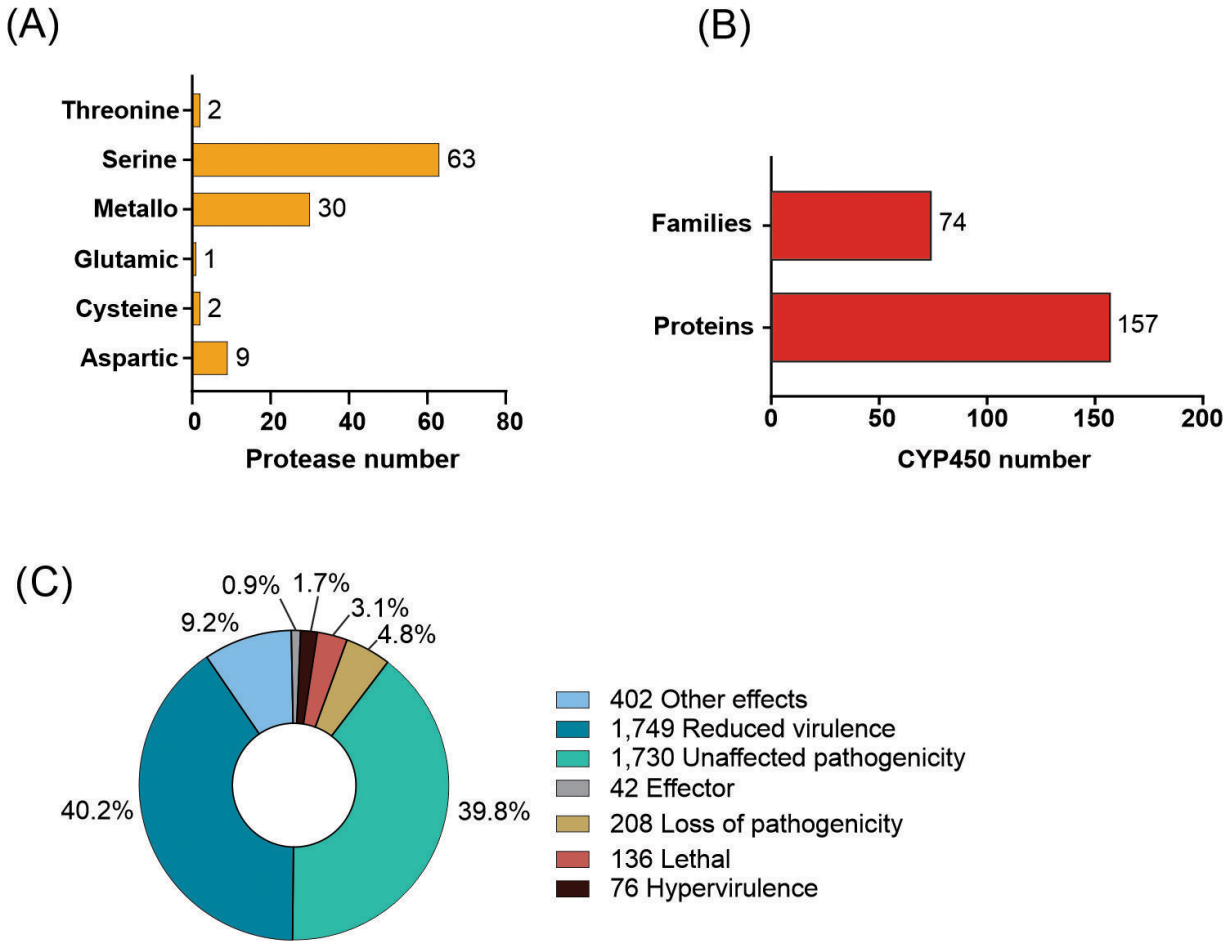
Number of putative proteins identified in the *Colletotrichum lindemuthianum* genome. **(A)** Putative proteases in the secretome, **(B)** Genes encoding proteins associated with pathogenicity of different categories, which have functionally characterized homologues in the manually curated Pathogen-Host Interaction (PHI-base) database, **(C)** putative cytochrome P450 (CYP450) proteins.

Using BLASTp against the MEROPS database, it was possible to identify 107 putative proteases in the *C. lindemuthianum* secretome (**Figure 2A**). Serine-proteases are the largest group with 63 proteins, followed by metallopeptidases with 30 proteins, which correspond to almost 87% of the total secreted proteases. In addition, 19 putative proteins that showed similarity with non-peptidases and two proteins considered protease inhibitors were identified in the secretome.

### 3.5 Cytochrome P450s

A total of 157 putative proteins with similar proteins in the cytochrome P450 database were identified (**Figure 2B**). These proteins were distributed among 74 CYP450 families. The CYP65 family has the most putative protein members, with 21 putative proteins, followed by the CYP68 family with 9 members, corresponding to 19% of putative CYP450 proteins. CYP65 and CYP68 are related to secondary metabolism. Most families (45 families) comprise one putative protein.

### 3.6 Sequences encoding proteins related to pathogenicity

In *C. lindemuthianum* 4,343 amino acid sequences with similarity to proteins in the PHI database were identified (**Figure 2C**), with most of the similar proteins having demonstrated effects on pathogenicity in other pathogens. While almost 40% of the identified putative proteins showed similarity with other proteins with no effect on pathogenicity. Of the 4,343 putative proteins, 1,749 were associated with reduced virulence, 208 with loss of pathogenicity, 76 with hypervirulence, and 42 with effector functions in the related pathogens.

### 3.7 Effector candidates

Previously, 349 candidate effector genes were found in the genome of *C. lindemuthianum* (Queiroz et al., 2019). The analysis was redone using Predector, an automated and combinative method for the predictive ranking of candidate effector proteins (Jones et al., 2021), and compared to the previous analysis (**Supplementary Figure S1**). This new prediction revealed a slight variation in the number of effector candidates; 373 were found originally, while the new assembly increased the number of effector candidates by nearly 1.4X (525 effectors). Alignment comparison showed that 262 of the candidate effector proteins from Queiroz et al. (2019) were similar to the newly predicted effectors, with 183 proteins sharing 100% similarity (**Supplementary Table S2**). In addition, all the effector candidates in the current assembly were found to be putatively secreted, being 45% (n= 238) cytoplasmic effectors, 50% (n= 264) apoplastic effectors, and 28% (n= 148) dual-localized effectors (apoplastic/cytoplasmic). Most effector candidates had no similarities to proteins in the PHI-base database and were predicted to be subcellularly localized to the nucleus (n= 78), chloroplast (n= 43), and mitochondria (n= 16). Information on the effector candidates may be found in **Supplementary Tables S3** and **S4**.

### 3.8 Chitinase-like proteins

**Figure 3:**
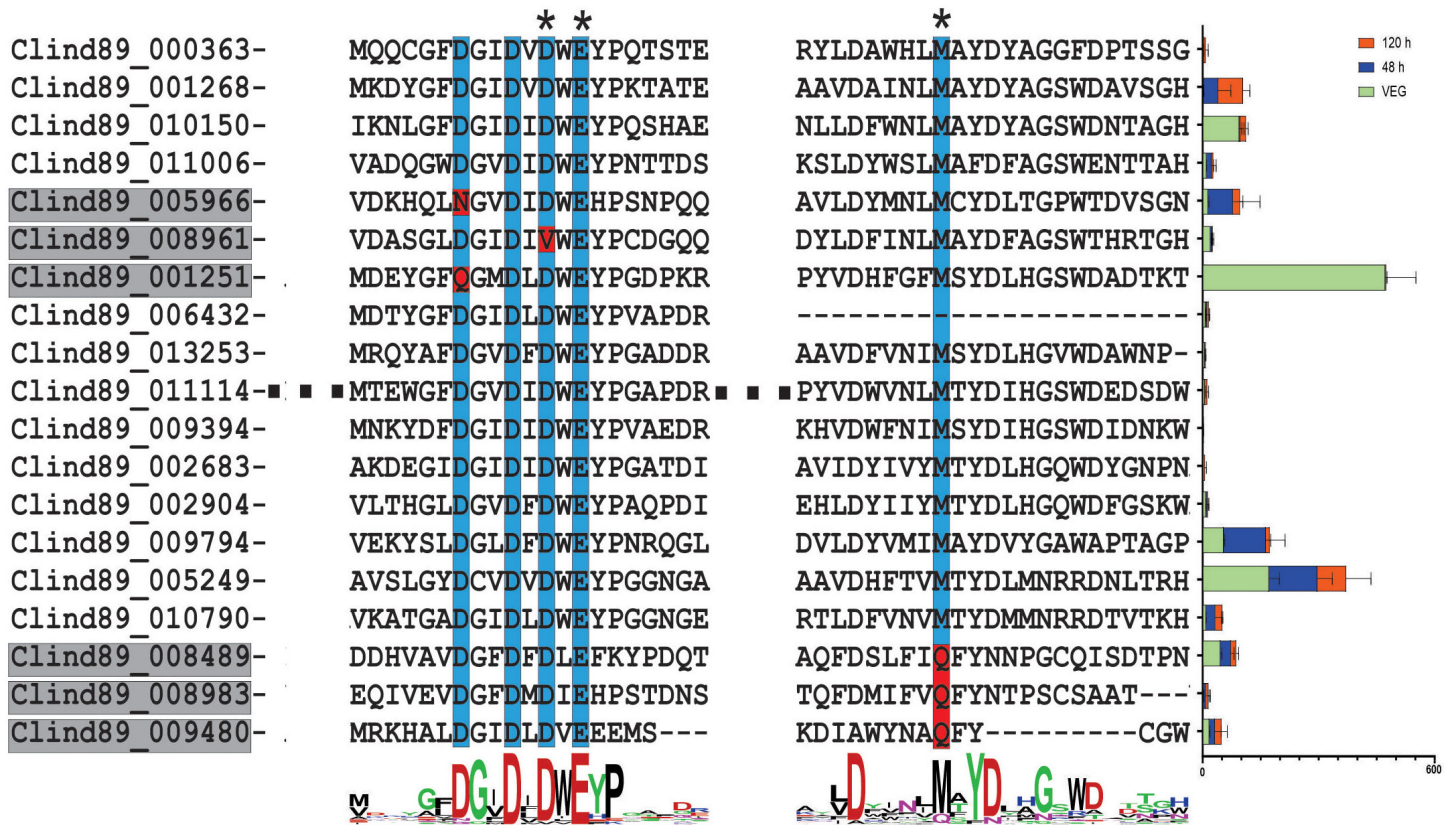
The *Colletotrichum lindemuthianum* chitinase-like proteins. The catalytic site of chitinase is DxDxE. Conserved amino acids are indicated by the blue color. Amino acid substitution in conserved regions is highlighted in red. Asterisk (*) indicates that amino acid substitution at the position results in loss of catalytic activity of the enzyme (Fiorin et al., 2018). At the end of each amino acid sequence, the expression of genes encoding chitinases (FPKM) at two stages of the *C. lindemuthianum-Phaseolus vulgaris* interaction (48 h and 120 h) and grown in GPYECH culture medium (VEG) is represented.

Some chitinase-like proteins can act as effectors, when harboring mutations in their catalytic motifs along with high expression of their encoding genes during the initial phases of a plant-pathogen interaction (Fiorin et al., 2018). Based on this information, a search was carried out to identify chitinase-like proteins in *C. lindemuthianum*. As a result, 18 putative proteins similar to chitinases were identified (**Figure 3**). Of these putative proteins, three were mutated in one amino acid present in the enzyme catalytic motif (xDxxDxDxE), with the replacement of aspartic acid (D) by asparagine (N), glutamine (Q), or valine (V). Three other putative proteins had a methionine (M) substitution for glutamine (Q) at a conserved residue. These mutated enzymes may have lost chitinolytic activity. Eight genes that encode chitinase-like proteins were expressed in the biotrophic phase of the host-pathogen interaction. Of the six mutated genes, three were expressed in the biotrophic phase.

### 3.9 Comparative genomic analysis

While certain gene groups related to pathogenicity have been analyzed in *C. lindemuthianum*, specific groups have been selected for comparative analysis, offering a broader perspective on the representation of these genes in comparison to other phytopathogenic fungi. Overall, comprehending the diversity and functionality of transporters, NLPs, SM gene clusters, TEs, and RIP mutations through comparative genomic analysis can yield insights into genome dynamics, evolution, and adaptation in phytopathogenic fungi.

#### 3.9.1 Transporters

**Figure 4:**
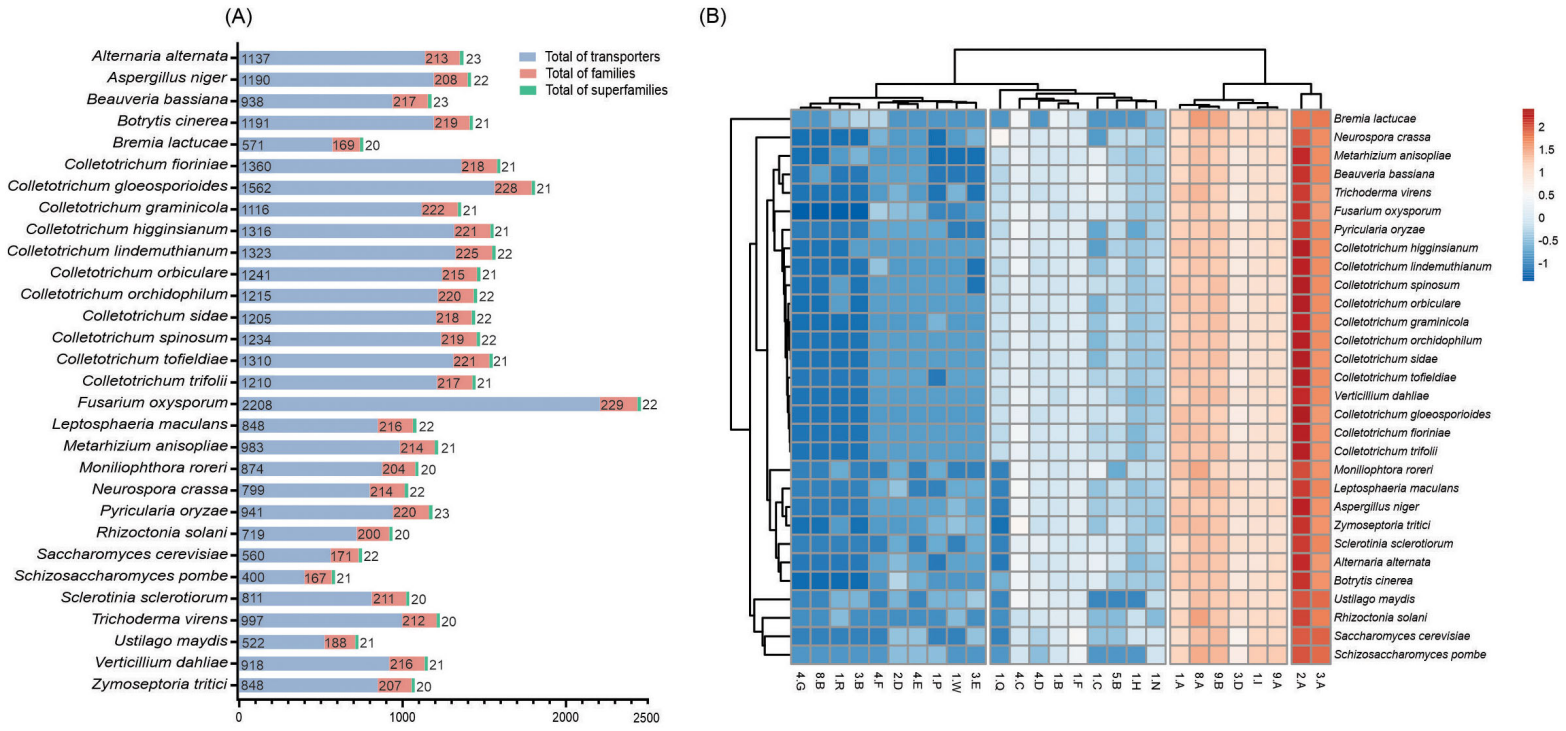
Repertoires of transporter proteins in the fungal genomes. **(A)** The comparative analysis of transporters, families, and superfamilies transporters in different fungal predicted proteomes. **(B)** Distribution of superfamilies transporters in different fungal predicted proteomes. Original values are ln(x)-transformed. Rows are centered and clustered using correlation distance and average linkage, while columns are clustered using Euclidean distance and average linkage.

*C. lindemuthianum* possesses a considerable number of transporters, totaling 1,323 putative proteins. Among the fungi studied, *Fusarium oxysporum* exhibited the highest number (n = 2,208), while *Ustilago maydis* encoded the fewest transporters (n = 522). These transporters were categorized into an average of 233 families and 23 superfamilies (**Figure 4**). Across *Colletotrichum* species, a consistent range of transporter numbers was noted, varying from 1,116 in *C. graminicola* to 1,562 in *Colletotrichum gloeosporioides*. Remarkably, fungal genomes encode for three major superfamilies, the major facilitator superfamily (MFS) (2.A.1), the ATP-binding Cassette (ABC) Superfamily (3.A.1), and the Membrane Fusion Protein (MFP) Family (8.A.1). These transporters are key classes that include uniporters, symporters, and antiporters involved in nutrient uptake and ion movement (Tanford, 1983; Forrest et al., 2011). They represent about 46, 15, and 7% of the transporter repertoire in *C. lindemuthianum*, respectively. In general, the Mitochondrial Carrier (MC) Family and the Amino Acid-Polyamine-Organocation (APC) Family were those with the most members in the MFS superfamily, followed by the H^+^ or Na^+^-translocating NADH Dehydrogenase (NDH) family in the ABC superfamily and the Voltage-gated K^+^ Channel Î²-subunit (KvÎ²) family in the MFP superfamily.

#### 3.9.2 Necrosis and ethylene-inducing peptide 1 - like proteins

**Figure 5:**
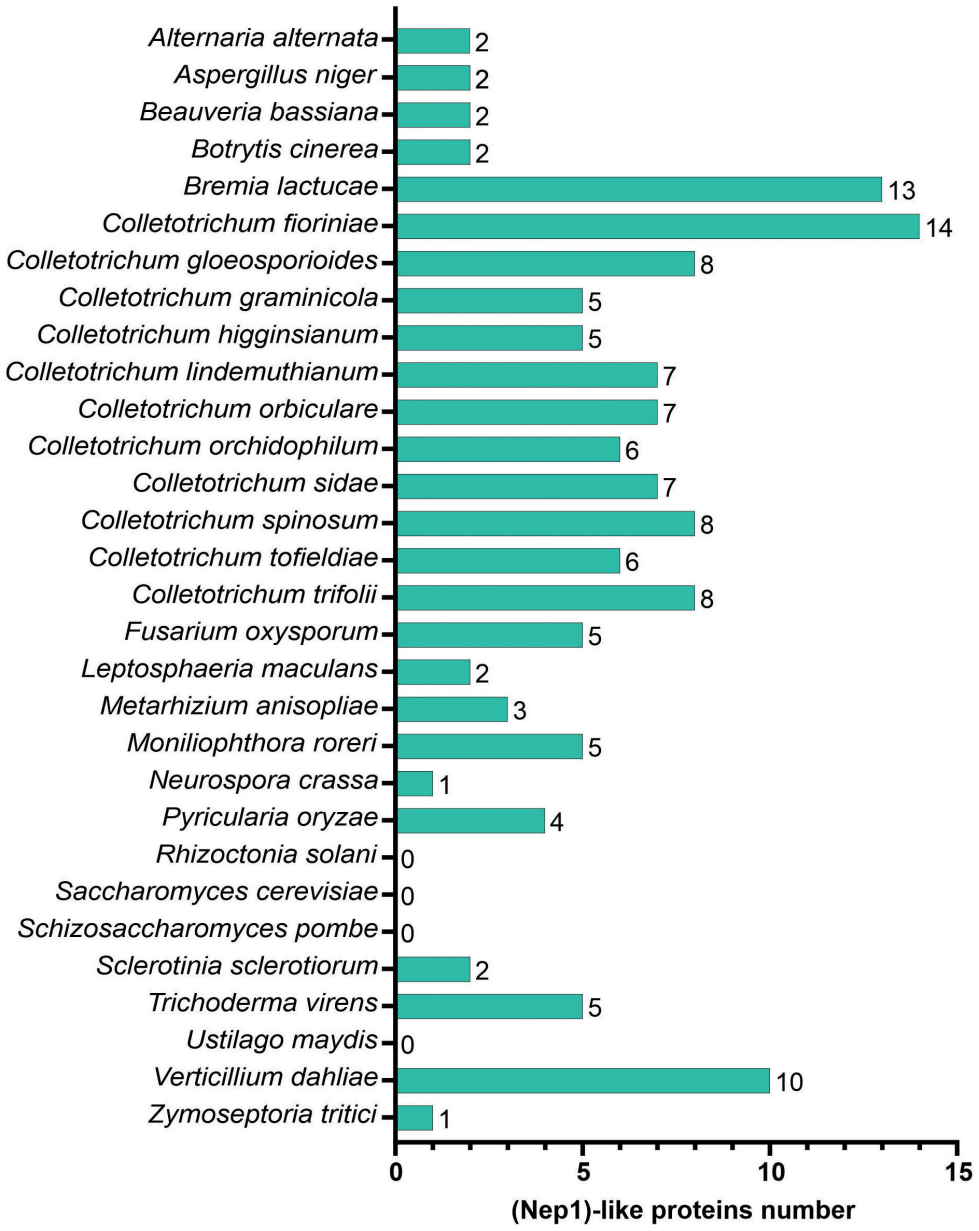
The comparative analysis of necrosis and ethylene-inducing peptide 1. (Nep1)-like proteins (NLPs) in different fungal predicted proteomes.

NLPs are present in microorganisms from different taxa that adopt diverse lifestyles (**Figure 5**). The number of putative NLPs in the fungi analyzed ranged from zero to 14. Seven putative NLPs were identified in *C. lindemuthianum*. The number of putative NLPs in other *Colletotrichum* species ranged from five to 14. Thirteen of the 30 analyzed fungi showed from zero to three NLP. The fungi with few NLPs were the basidiomycetes *Rhizoctonia solani* and *U. maydis*, and the ascomycetes yeasts *Saccharomyces cerevisiae* and *Schizosaccharomyces pombe* with no NLPs. Those fungi with the greatest number of NLPs were the ascomycetes *Verticillium dahliae*, *Colletotrichum fioriniae*, and the oomycete *Bremia lactucae* with 10, 14, and 13 NLPs, respectively.

**Figure 6:**
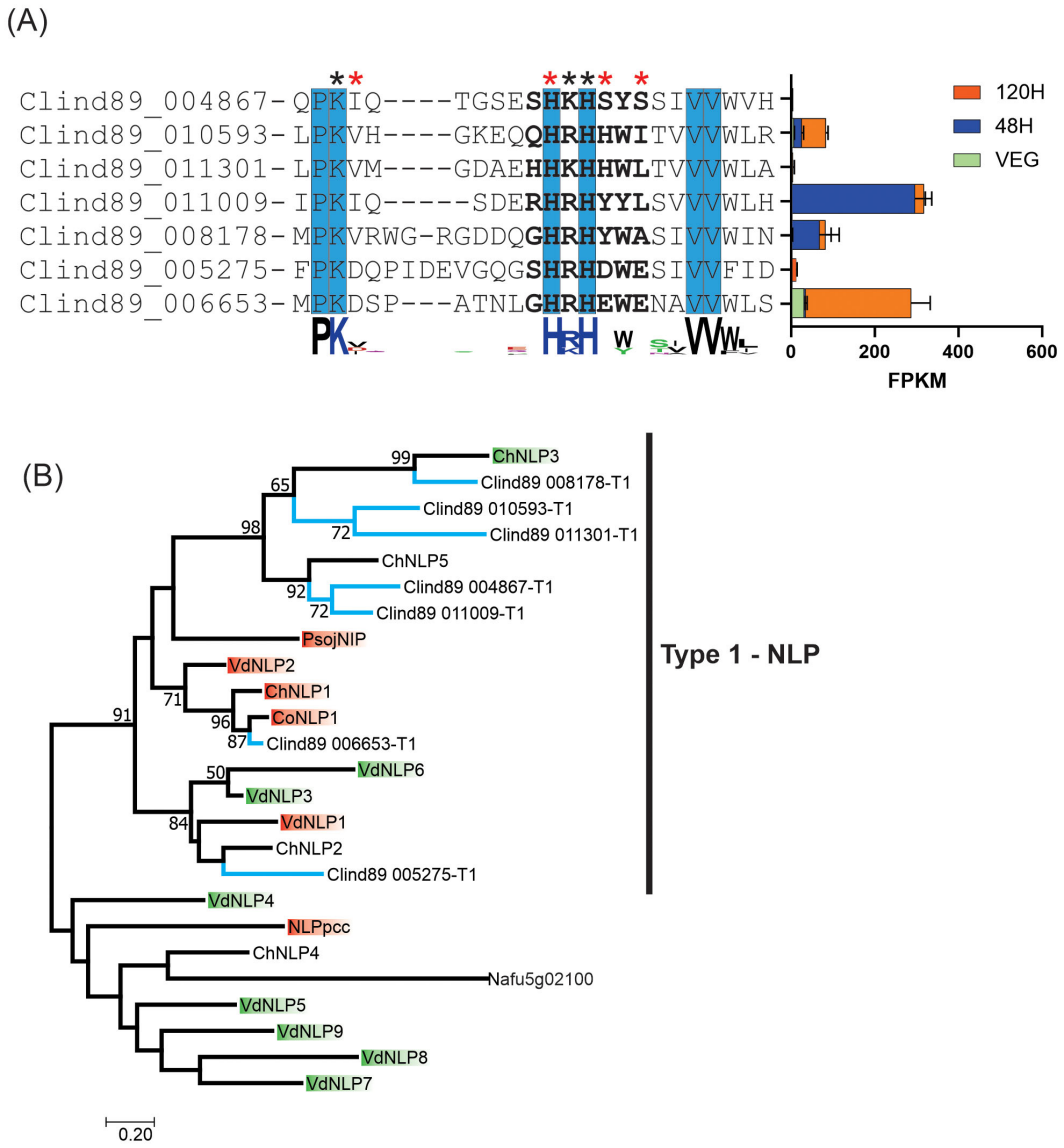
The *Colletotrichum lindemuthianum* necrosis and ethylene-inducing peptide 1 (Nep1)-like proteins (NLPs) and phylogenetic reconstruction of NLPs. **(A)** The conserved characteristic motif of NLPs is shown in bold (GHRHDWE). Invariant amino acids near or in the GHRHDWE motif are highlighted in blue. Red asterisks (*) indicate that substitution of amino acids at the position causes abolition in necrosis activity and black asterisks indicate reduction (Ottmann et al., 2009). At the end of each amino acid sequence, the expression of genes encoding NLPs (FPKM) in two stages of the *C*. *lindemuthianum-Phaseolus vulgaris* interaction (48 h and 120 h) and grown in GPYECH culture medium (VEG) is represented. **(B)** Blue branches indicate the NLPs of *C*. *lindemuthianum* ChNLP1. Red boxes indicate necrosis activity while green boxes indicate absence. ChNLPs are from *Colletotrichum higginsianum* (Kleemann et al., 2012), CoNLP1 from *Colletotrichum orbiculare* (Azmi et al., 2018), VdNEPs from *Verticillium dahliae* (Zhou et al., 2012), PsojNIP (type 1) from *Phytophthora sojae* (Qutob et al., 2002), NLPpcc (type 2) from *Pectobacterium carotovorum* subsp. *carotovorum* (Mattinen et al., 2004) and Afu5g02100 (type 3) from *Aspergillus fumigatus* (Upadhyay et al., 2012). Maximum Likelihood phylogenetic analysis was performed with JTT matrix-based substitution model and 10,000 replicates of bootstrap.

The seven putative NLPs in *C. lindemuthianum*, all had a mutated GHRHDWE conserved motif (**Figure 6**). The motif was 85% conserved in two putative proteins (Clind89-006653-T1 and Clind89-005275-T1). All *C. lindemuthiaum* putative NLPs had the conserved Histidine (H) at the same position as the consensus motif. Amino acid substitution at some position in the GHRHDWE motif may cause abolition or reduction in necrosis activity (Ottmann et al., 2009). Only the Clind89-005275-T1 putative protein had no mutation in any of the three amino acid positions that when mutated may cause necrosis activity abolition. The gene encoding Clind89-006653-T1 had the highest genic expression at 120 h post inoculation during the *C. lindemuthianum-Phaseolus vulgaris* interaction, corresponding to the necrotrophic phase of the disease.

A phylogenetic reconstruction of three NLP types from fungi related to *C. lindemuthianum* (*Colletotrichum higginsianum*, *C. orbiculare*, and *V. dahliae*) showed a clade including all NLPs from *C. lindemuthianum* together with type 1 NLP (PsojNIP) from *P. sojae* (**Figure 6**). Some of the NLPs used for phylogenetic reconstruction were analyzed for their capability to cause necrosis (Qutob et al., 2002; Kleemann et al., 2012; Zhou et al., 2012; Azmi et al., 2018). The putative proteins Clind89 005275-T1 and Clind89-006653-T1 were grouped with one and three proteins with a proven ability to induce necrosis, respectively. These putative proteins had the highest motif identity, and the gene encoding one of them had the highest genic expression.

#### 3.9.3 Secondary metabolite gene clusters

**Figure 7:**
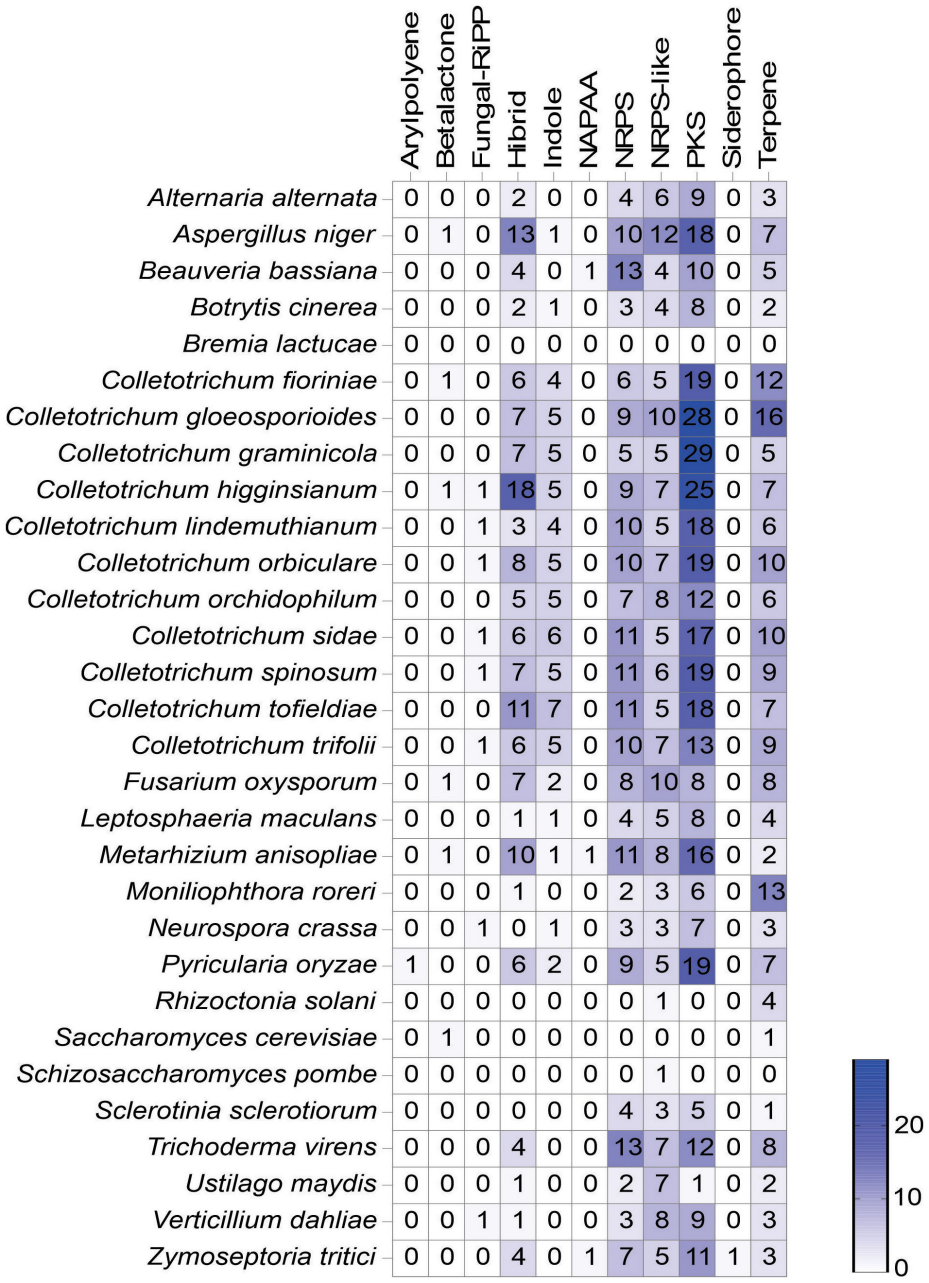
Distribution of secondary metabolite (SM) gene clusters predicted in different fungi using antiSMASH (fungal version). The numbers inside the boxes represent the number of secondary metabolite clusters found of each type, and the color intensity is related to the number. NRPS, Non-ribosomal peptide synthetases; PKS, Polyketide synthases; RiPP, Ribosomally synthesized and post-translationally modified peptide; NAPAA, Non-alpha poly-amino group acids.

A total of 47 SM clusters were predicted in the *C. lindemuthianum* genome (**Figure 7**). Most SM clusters were Polyketide synthetases (PKSs) and Nonribosomal Peptide Synthetases (NRPSs). SM clusters for terpene and indole production were also identified. Out of the 10 putatively identified NRPSs, two related to chrysogine and dimethylcoprogene production displayed 100% similarity with described clusters, whereas those related to aspirochlorine and apicidin production showed less than 30% (**Supplementary Table S5**). Among the 18 T1-PKSs, only the cluster related to alternapyrone production demonstrated 100% similarity, while those related to naphtalene, cercosporin, and monacolin K production exhibited less than 35%. Among the 5 NRPS-Like, only the cluster related to EQ-4 recorded 100% similarity, while the cluster related to biotin showed 66%. In the case of the 6 terpenes, only a cluster related to squalestatin S1 production was found, with 40% similarity. No known biosynthetic clusters were found for the fungal-RiPP, hybrid, and indole production.

The number of SM clusters in the genomes of the *Colletotrichum* species ranged from 43 for *Colletotrichum orchidophilum* to 75 for *C. gloeosporioides*. Among the species belonging to the orbiculare complex (*C. lindemuthianum*, *C. orbiculare*, *C. sidae*. *C. spinosum* and *C. trifolii*), *C. lindemuthianum* showed the fewest number of SM clusters. The fungi from the *Colletotrichum* genus presented a greater number of SM clusters compared to all 30 microorganisms analyzed. The greatest number of SM clusters was identified in *C. gloeosporioides* (n= 75), and the lowest in *B. lactucae* (n= 0). The same type of SM clusters were observed in the genome of most fungi analyzed, mainly PKS, NRPS, indole and terpene clusters. Most fungi presented some hybrid SM clusters (**Supplementary Figure S2**). *Colletotrichum lindemuthianum* had the fewest hybrid SM clusters among the fungi from *Colletotrichum* species, and the *C. higginsianum* had the greatest number among all fungi analyzed, 3 and 18 respectively. The most common hybrid SM clusters were a combination of NRPS and PKS type 1.

#### 3.9.4 Transposable elements and repeat-induced point mutations

**Figure 8:**
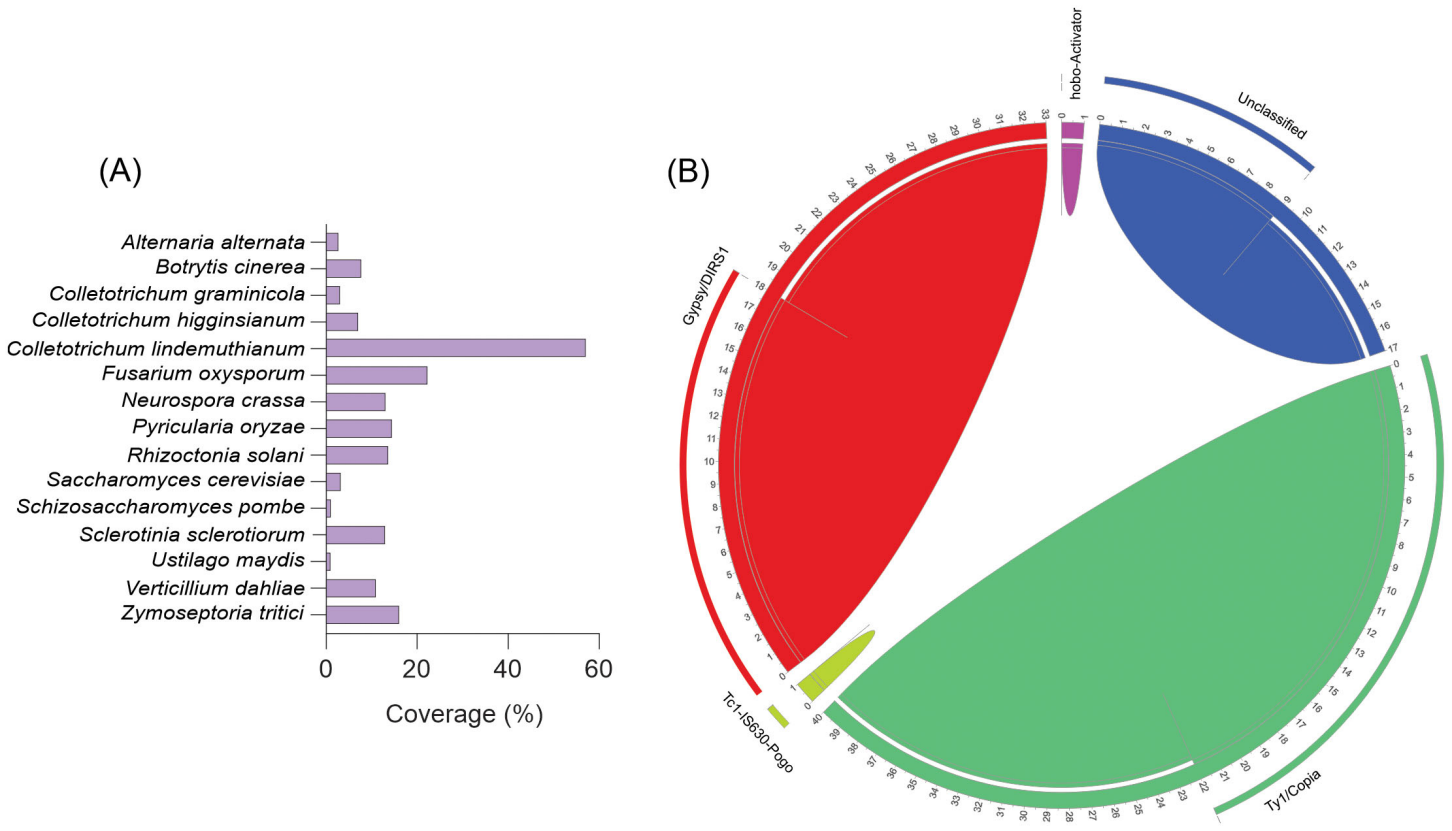
Repetitive elements among different fungal species. **(A)** The comparative analysis of repetitive elements in different fungi. **(B)** Repetitive elements in *Colletotrichum lindemuthianum*. The chord diagram illustrates the distribution and relationships of various transposable element families. The outer chord around the circle represents the percentage of each element family. The inner chords depict the proportion of these elements compared to the total elements found in the genome.

The diversity and abundance of repetitive elements in the *C. lindemuthianum* genome was investigated. Comparisons with metrics obtained from other fungal genomes that had been sequenced with a long-read sequencing strategy similar to that used in the present study were made (**Figure 8**). Our analysis revealed that 60% of the *C. lindemuthianum* genome comprises repetitive elements, a significantly higher proportion compared to other fungi (**Figure 8A**). Specifically, this exceeds the repetitive element content of *A. alternata* by over 30 times and that of its close relative, *C*. *higginsianum*, by nine times. The comparative genomic analysis of seven *Colletotrichum* species revealed a significant variation in total transposable element (TE) content, ranging from 4.3% to 44.8% of the genome in *Colletotrichum scovillei* to *C*. *orbiculare*, respectively (Rao et al., 2018). Hence, the observed differences in TE numbers may shed light on why *C. lindemuthianum* possesses one of the largest genomes within its genus.

Upon closer examination of the classes of repetitive elements present, we found that a significant portion of *C*. *lindemuthianum’s* genome comprises retroelements, particularly the *Ty1/Copia* superfamily, which accounts for 22% of the genome and represents 40% of the repetitive elements. Additionally, the *Gypsy/DIRS1* superfamily is notable by its presence. Furthermore, DNA transposons, specifically *Tc1-IS630-Pogo*, constitute approximately 1% of the genome, with 9% of these elements remaining unclassified (**Figure 8B**).

*Colletotrichum lindemuthianum* stands out for having a remarkably high proportion of its genome affected by RIP (54.77%), the highest among all genomes evaluated, indicating substantial RIP activity within this species (**Table 2**). Although the other members of the orbiculare complex analyzed (*C. orbiculare*, *C. sidae*, *C. spinosum*, *C. trifolii*) also have large genomes, they were not as affected by RIP as *C. lindemuthianum*. Apparently, RIP contributes significantly to the low GC content in the *C. lindemuthianum* genome. The RIP index values were similar to those observed in other species of the genus *Colletotrichum* and even *Neurospora crassa* (**Supplementary Table S6**).

**Table 2:** RIP profile in different fungi.

Species	Genome Size	GC content (%)	RIP affected genomic (%)
*Alternaria alternata*	33 Mb	51,4	0,31
*Aspergillus niger*	34 Mb	50,35	0,07
*Beauveria bassiana*	34 Mb	51,51	2,88
*Botrytis cinerea*	43 Mb	42	0,49
*Colletotrichum fioriniae*	50 Mb	51,91	2,79
*Colletotrichum gloeosporioides*	62 Mb	50,83	7,75
*Colletotrichum graminicola*	52 Mb	49,11	12,69
*Colletotrichum higginsianum*	51 Mb	54,41	3,34
*Colletotrichum lindemuthianum*	100 Mb	37,25	54,77
*Colletotrichum orbiculare*	90 Mb	37,57	22,62
*Colletotrichum orchidophilum*	49 Mb	51,07	5,26
*Colletotrichum sidae*	87 Mb	37,96	19,25
*Colletotrichum spinosum*	83 Mb	38,71	20,46
*Colletotrichum tofieldiae*	53 Mb	53,28	3,51
*Colletotrichum trifolii*	110 Mb	36,62	25,27
*Fusarium oxysporum*	48 Mb	47,63	2,61
*Leptosphaeria maculans*	45 Mb	45,19	11,84
*Metarhizium anisopliae*	43 Mb	50,9	1,37
*Moniliophthora roreri*	57 Mb	46,51	0,22
*Neurospora crassa*	41 Mb	48,23	15,26
*Pyricularia oryzae*	41 Mb	51,61	0,36
*Rhizoctonia solani*	41 Mb	47,63	0,52
*Saccharomyces cerevisiae*	13 Mb	36,05	0,07
*Schizosaccharomyces pombe*	12 Mb	38,15	0,16
*Sclerotinia sclerotiorum*	38 Mb	41,8	0,45
*Trichoderma virens*	39 Mb	49,25	1,16
*Ustilago maydis*	20 Mb	54,03	0,01
*Verticillium dahliae*	34 Mb	55,79	1,76
*Zymoseptoria tritici*	40 Mb	52,14	15,3

The homologous sequence of MAT1-2-1 from *C. lindemuthianum* isolate MU 03 (Garciía-Serrano et al., 2008) was identified in the genome of *C. lindemuthianum* A_2_ 2-3, with over 99% coverage and identity, but no homologous sequences were found for the MAT1-1 sequences from *F. graminearium*, as has already been observed in other genomes of *Colletotrichum* spp. analyzed. Although RIP is associated with the sexual cycle, this cycle is not frequently observed in *C. lindemuthianum* and is not subject to the control of a traditional mating-type system, since both idiomorphs were not found in all isolates analyzed to date.

## 4 Discussion

The genome of *C. lindemuthianum* isolate A_2_ 2-3 physiological race 89 presented in this study is one of the largest genomes reported for the *Colletotrichum* genus. Considering the genome complexity of this fungus due to its high number of repetitive elements and larger size than most fungi of the same genus, it was possible to obtain a high-quality 100 Mpb genome containing 119 scaffolds.

In eukaryotic organisms with small genomes, the genome size and gene number are commonly associated (Raffaele and Kamoun, 2012). The *C. lindemuthianum* genome presented here increased the number of predicted genes compared to the previous assembly (Queiroz et al., 2017). However, there is no correlation between the genome size and gene number for fungal genomes of the *Colletotrichum* genus. In addition, the *C. lindemuthianum* genome comprises more than 60% of A+T-rich regions. This low G+C content (%) and gene density are indicators that *C. lindemuthianum* genome size expansion may have followed the “mutation-hazard” model (Kelkar and Ochman, 2012), where genome expansion would be driven by genetic drift, allowing mobile element accumulation, which could contribute to the existence of genomic regions with low G+C content (%).

Carbohydrate-active enzymes (CAZymes) have central roles for degradation of polysaccharides and oligosaccharides in plants, and there were many CAZymes in *C. lindemuthianum*, with more than 40% of the CAZymes predicted to be secreted. Carbohydrate degradation is one of the main strategies of phytopathogenic fungi to enable them to establish themselves in host plants and cause disease (Schmitz et al., 2019). In *Colletotrichum*, CAZymes are mainly xylanases, pectinases, and cellulases (Gan et al., 2012). The enzymes involved in *C. lindemuthianum* plant cell wall degradation include a higher proportion of pectinases compared to cellulases and xylanases, with many of these enzymes directly contributing to the fungus’s pathogenicity in plants. In *C. lindemuthianum*, the expression of many genes encoding pectinases during the fungus-host interaction has already been shown (Silva et al., 2022), and the deletion of some of these genes may cause reduction in pathogen aggressiveness (Cnossen-Fassoni et al., 2013). The pectinases produced by *C. lindemuthianum* are necessary for the maceration of common bean tissue in the necrotrophic phase, but they also play a crucial role in a stage preceding the biotrophic phase, as has been demonstrated for the endopolygalacturonase PLPG2, which associates with the cell wall, possibly reducing severe cellular damage that could trigger defense responses (Herbert et al., 2004). Therefore, it is evident that CAZymes can play a central role in fungal pathogenicity caused by *Colletotrichum* species.

Proteases are enzymes that degrade protein into amino acids and peptides, being implicated in the pathogenicity of many phytopathogenic fungi such as *Colletotrichum* species (Redman and Rodriguez, 2002). The predicted secretome of *C. lindemuthianum* contains 107 proteases, mainly serine and metallopeptidases, such as observed for other *Colletotrichum* species (Rao and Nandineni, 2017). There are more proteases encoded by the genomes of *Colletotrichum* species than by other fungal pathogens (Gan et al., 2012), and a study with *Colletotrichum coccodes* demonstrated a relationship between proteases and plant mortality and symptoms induction, suggesting that these enzymes play a fundamental role in pathogenicity and virulence (Redman and Rodriguez, 2002). Although some studies have investigated *C. lindemuthianum* proteases, little is known about the role of these enzymes in this fungus’s pathogenicity (Wijesundera et al., 1989). However, the data revealed about these enzymes after sequencing may serve as a guide for future studies. The characterization of both CAZymes and proteases of *C. lindemuthianum*, presented here, may help to elucidate the mechanisms by which the fungus invades its host and establishes pathogenesis.

In the *C. lindemuthianum* genome, 157 genes encoding for cytochrome P450s (CYP450s) were found and distributed in 74 families, with CYP65 and CYP68 being the families with the most putative proteins. CYP450s are oxidative enzymes widely distributed in living organisms, including fungi (Moktali et al., 2012). In phytopathogenic fungi, CYP450s play critical roles in the detoxification of toxic compounds produced by the host and in secondary metabolite synthesis, activities which are essential for pathogenicity and virulence (Črešnar and Petrič, 2011). In the fungus *Colletotrichum siamense*, a cytochrome P450-encoding gene was proven to be related to fungal sensitivity for the toxic compound fludioxonil by genetic manipulation (Guan et al., 2022). Many CYP450s genes in the *C. lindemuthianum* genome are related to secondary metabolite production (Moktali et al., 2012). PHI-base is a manually curated database that gathers information on validated genes related to the pathogenicity of fungi and other pathogens (Urban et al., 2015). Putative proteins from *C. lindemuthianum* were similar to proteins in PHI-base that affected pathogenicity, that when mutated resulted in virulence reduction, pathogenicity loss, and hypervirulence, and had effector functionality. The information from the PHI-base analysis could be used to determine candidates for further research and could help identify possible targets for developing more specific and effective fungicides.

In this study, the new genome assembly of *C. lindemuthianum* revealed 525 effector candidates, a slight increase from the previous count of 349 (Queiroz et al., 2019). Alignment comparison showed 262 candidate effector proteins from the prior study were similar to the new predictions, with 183 sharing full similarity. The effector candidates were predominantly putatively secreted, including cytoplasmic, apoplastic, and dual-localized effectors. Most candidates lacked similar poteins in the PHI-base database, and various subcellular localizations were predicted, such as the nucleus, chloroplast, and mitochondria. Overall, this study indicates a refinement in the identification of effector candidates in *C. lindemuthianum* and provides insights into their potential roles, secretion, and subcellular localization. The increase in effector candidate counts and the findings about their predicted localization and interactions with host organisms could have implications for understanding the pathogenic mechanisms and developing strategies for disease management.

In *C. lindemuthianum*, 18 sequences encoding proteins are similar to chitinases and six present mutations in conserved regions. Chitinase-like proteins differ from classical chitinases because they do not hydrolyze chitin due to mutations in some conserved amino acids (Fiorin et al., 2018). When analyzing the interaction of another *C. lindemuthianum* isolate, transcripts of chitin-like protein genes were observed at 24, 48, and 72 hours post-inoculation, corresponding to the biotrophic phase of the pathogen (Romero et al., 2024). These *C. lindemuthianum* putative proteins may also have lost their chitinolytic activity and remain capable of only binding to chitin, thus acquiring a new function as effector proteins, preventing the elicitation of plant defense responses. A similar situation was observed in *Moniliophthora* spp., where chitinase-like proteins presented mutations in conserved sites and lost their chitinolytic activity but the encoding genes were highly expressed during the biotrophic phase (Fiorin et al., 2018). It was suggested that these proteins function as effector proteins, sequestering free fragments of chitin from the pathogen and preventing recognition by the host. These proteins play an interesting role in the pathogenicity of these organisms, by manipulating the host plant’s immune system.

There were a greater number of transporters in the genome of *C. lindemuthanum* compared to the other fungi analyzed. The main transporters are members of the MFS and ABC superfamilies. The transition to necrotrophy in pathogenic fungi leads to a significant shift in the expression of genes encoding hydrolases, proteases, and transporters, which collectively facilitate the degradation of plant tissues and the assimilation of intracellular nutrients (Gan et al., 2012; O’Connell et al., 2012; Alkan et al., 2015; Zhang et al., 2018). Nutrients liberated from the infected plant tissue are subsequently absorbed through transmembrane transporters, including oligopeptide transporters, ABC transporters, and members of the MFS. Some of these transporters have been implicated in the pathogenicity of *Colletotrichum* spp. with documented roles in sugar transport, oxidative stress resistance, and pathogenesis (Chagué et al., 2009; Shnaiderman et al., 2013; Liu et al., 2017). Similarly, comparative genome analyses across the *Colletotrichum* genus unveiled tailored transporter profiles, often associated with the MFS type transporters, encompassing myo-inositol transporters, general glucose transporters, and multiple members of the anion:cation symporter (ACS) family (Gan et al., 2016). In addition, within *C. lindemuthianum*, our study revealed a prominent representation of the MFS, ABC, and MFP superfamilies, collectively constituting a substantial portion of the fungal transporter repertoire The results obtained with the isolation of mutants (Δ*abcCl1*) demonstrate that the AbcCl1 transporter protects *C. lindemuthianum* from toxic compounds produced by the common bean, probably in the biotrophic phase of the fungus (Oliveira et al., 2022). In addition, transporters should also be important for the export of toxins during the necrotrophic phase. Notably, the deletion of specific genes encoding these transporters has also been associated with the role of hexose transporters in the capture of carbon sources during the necrotrophic phase of the plant-pathogen interaction (Pereira et al., 2013).

NLPs are conserved proteins presenting a seven amino acid motif (GHRHDWE), they induce ethylene production and necrosis in leaves of dicot plant species (Oome and Van Den Ackerveken, 2014). In this study, the majority of microorganism genomes analyzed contained at least one NLP. It described three types of NLP in different eukaryotic and prokaryotic microorganisms, pathogenic or not for plants (Seidl and Van den Ackerveken, 2019). *Colletotrichum lindemuthianum* has a similar number of NLPs to most other *Colletotrichum* species, and these NLPs seem to be all from type 1. Type 1 NLP is mainly found in microorganisms that interact with plants (Oome and Van Den Ackerveken, 2014). In previous studies genomes from the *Colletotrichum acutatum* species complex (*Colletotrichum nymphaeae*, *Colletotrichum simmondsii*, *Colletotrichum fioriniae*, and *Colletotrichum salicis*) have a greater number of NLPs, almost double that of the others *Colletotrichum* species (Baroncelli et al., 2016), and some *Colletotrichum* species may have different types of NLP (Liu et al., 2020). In the obligate biotrophic oomycete *Plasmopara viticola* genome eight NLPs were identified, and their encoding genes were expressed at different stages of the pathogen-host interaction, but none of them were able to cause necrosis in *Nicotiana benthamiana* when transiently expressed (Schumacher et al., 2020; Askani et al., 2021).

In *C. lindemuthianum*, the seven putative NLP identified had variations in the heptamer consensus motif and encoding genes had different expression profiles. Previously mutant NLP proteins with amino acid alterations in the conserved consensus motif ‘‘GHRHDWE” had their activity reduced or lost (Ottmann et al., 2009). In *C. higginsianum*, similar results were observed (Kleemann et al., 2012). The ChNLP1 NLP was expressed at the switch to necrotrophy in the interaction of *C. higginsianum* with *N. benthamiana* and ChNLP3 was expressed in appressoria before penetration. When these proteins are transiently expressed in *N. benthamiana*, ChNLP1 was able to induce cell death, whereas ChNLP3 was not. The ChNLP3 had a mutation at critical amino acids that resulted in loss of function whereas, ChNLP1 had not. We observed that the *C. lindemuthianum* Clind89-006653-T1 putative protein is very similar (92% of identity) to the *C. orbiculare* CoNLP1 protein, and both had the same amino acid substitution in the motif GHRHDWE. Despite the mutation, the *C. orbiculare* CoNLP1 protein can induce necrosis in *N. benthamiana* when transiently expressed via agroinfiltration (Azmi et al., 2018). In *C. orbiculare* an amino acid substitution in the critical motif of NLP1H127A reduced host specificity resulting in ability to cause necrosis only in melon, while the protein with a complete motif was capable of causing necrosis in melon and *N. benthamiana* (Chen et al., 2021). Based on sequence analyses of the conserved NLP motif, gene expression, and phylogenetic reconstruction, we hypothesize that only a few *C. lindemuthianum* NLPs could induce necrosis in the host, with Clind89-006653-T1 being prime candidate. This can be confirmed by isolation and analysis of mutants in future research.

Control of NLP gene expression is critical since recognition of NLPs may trigger host defenses (Azmi et al., 2018). An isolate of *C. obiculare* with constitutive NLP expression had a reduced host range when measured against five typical hosts. In contrast, pathogen inoculation in *N. benthamiana* was able to cause disease. NLPs may be expressed during different phases of the pathogen-host interaction, but the gene is commonly expressed in the necrotrophic phase, where the NLP protein is considered a virulence factor (Seidl and Van den Ackerveken, 2019). Because of the importance of NLPs for several phytopathogenic microorganisms in the interactions with their hosts, the NLPs may be a target for developing novel agents and strategies for plant pathogen control (Pirc et al., 2021).

The genome of *C. lindemuthianum* contains a diverse range of secondary metabolite (SM) clusters, including NRPS, PKS, and those responsible for synthesis of indoles and terpenes. The number of SM clusters in *C. lindemuthianum* is fewer than in the majority of other *Colletotrichum* species, but greater than in most other analyzed genera. Ascomycetes exhibit in general a higher number of SM clusters compared to other fungal taxa (Collemare et al., 2008). The variable number of SM clusters found in different species, both within the *Colletotrichum* genus and across analyzed species, may be influenced by the fungi’s lifestyle or their interaction with the host. Also, this variation indicates a vast diversity of produced compounds, presuming a specific metabolite for each predicted cluster (Collemare et al., 2008; O’Connell et al., 2012). Fungal ecology may drive adaptations that result in the gain or loss of specific metabolite clusters, influenced by various environmental pressures (Wisecaver and Rokas, 2015). In phytopathogenic fungi, these metabolites are of great importance in plant interactions, facilitating host infection and colonization, and directly influencing disease development and fungal survival (Pusztahelyi et al., 2015; Moraga et al., 2019). Many of these metabolites are phytotoxic, such as the colletotrichins found in *Colletotrichum* spp., capable of inducing symptoms similar to the disease itself and damaging the host’s plasma membrane (Gohbara et al., 1978; Duke et al., 1992).

From the 47 SM clusters found in *C. lindemuthianum*, only a few were similar with known biosynthetic SM clusters. Among their products, chrysogine (NRP) is recognized as a yellow pigment produced by various fungi, including *Penicillium chrysogenum*. Although it does not exhibit antimicrobial properties, there are indications that it may play a role in the protection against ultraviolet (UV) radiation (Viggiano et al., 2018). Dimethylcoprogene (NRP) is classified as a siderophore, found in various fungi, and may be involved in iron uptake in *C. lindemuthianum*, as described in other phytopathogens such as *C. graminicola*, *A. alternata*, *Cochliobolus heterostrophus*, and *F. gramineaum* (Lee et al., 2005; Oide et al., 2006; Chen et al., 2013; Albarouki et al., 2014). Studies also suggest that siderophores play a relevant role in virulence and protection against oxidative stress responses (Lee et al., 2005; Oide et al., 2006; Chen et al., 2013; Albarouki et al., 2014).

In *C. lindemuthianum*, the alternapyrone biosynthesis cluster was identified, which is also present in *Parastagonospora nodorum*, a wheat pathogen. During the interaction between *P. nodorum* and wheat, an increase in expression of the genes in the cluster was observed during plant development, suggesting positive regulation. Beyond that, when alternapyrones were applied in wheat seed germination assays a phytotoxic effect was observed (Ipcho et al., 2012; Li et al., 2018). *Colletotrichum lindemuthianum* possesses the microperfuranone biosynthesis cluster (EQ-4, NRP) with 100% similarity to that in the *A. nidulans* genome (MIBig accession: BGC0001668). This molecule has antimicrobial activity against various microorganisms, such as *Edwardsiella ictarda*, *C. gloeosporioides*, *Vibrio harveyi*, and others (Lü et al., 2020).

As noted, the majority of SM clusters in *C. lindemuthianum* show no similarity to previously described SM clusters. This indicates that the functions of several products derived from these SM clusters remain unknown, underscoring the need for further investigations. These unidentified products may contribute to the pathogenicity of *C. lindemuthianum*, as demonstrated in *C. gloeosporiodes*, where secondary metabolites extracted from different isolates exhibited varying phytotoxic activities in yam (*Dioscorea* spp.) (Abang et al., 2009).

Different metabolites with varied functions are produced by *Colletotrichum* spp. during host plant infection (Moraga et al., 2019), and most of these metabolites are unknown. Considering that these metabolites may have functions such as helping to combat competitors (Arivudainambi et al., 2011) or contributing to plant tissue necrosis (Reveglia et al., 2023), the phase of the life cycle in which they are produced is related to the environment where the pathogen is found. Determining the ideal conditions for the expression of a given SM cluster remains a challenge, as is evaluating the metabolites produced during infection.

The new genome assembly of *C. lindemuthianum* comprises a high percentage of repetitive sequences. This represents the highest percentage of repetitive sequences reported for fungi within the *Colletotrichum* genus, surpassing figures for other species like *Colletotrichum lentis* (4.56%), *Colletotrichum truncatum* (6.8%), and *C. orbiculare* (8.3%) (Rao et al., 2018; Bhadauria et al., 2019). Among the repetitive sequences identified in the genome of *C. lindemuthianum*, Class I transposable elements (TEs) are the most abundant. Elements of the LTR order, belonging to the *Gypsy* and *Copia* superfamilies, are the most represented among these. These results corroborate findings in other species such as *C. truncatum*, *C. higginsianum*, *C. graminicola*, *C. scovillei*, *Colletotrichum chlorophyti*, and *C. orchidophilum* (Rao et al., 2018). Despite their phylogenetic proximity and having several similar genomic characteristics, the species *C. lindemuthianum* and *C. orbiculare* seem to exhibit differences in the distribution of their TEs. While the *Ty1/Copia* superfamily is one of the most abundant in the genomes of *C. lindemuthianum*, Rao et al. (2018) reported that elements from the *Copia* superfamily are the most abundant in *C. orbiculare*, followed by elements from the *Gypsy* superfamily. Furthermore, it was observed that the largest fraction of TEs in the *C. orbiculare* genome consists of unknown elements, which contrasts with the data observed in the present work for the species *C. lindemuthianum*, where it was possible to classify most of the identified elements.

The significant proportion of the genome of *C. lindemuthianum* consisting of repetitive elements, coupled with its status as the largest genome within the genus (Queiroz et al., 2017), presents an intriguing context for interpreting its observed RIP activity. To prevent potential damage by the movement of TEs into housekeeping genes, mechanisms such as DNA methylation (Goll and Bestor, 2005; Zemach et al., 2010), repeat-induced point mutation (RIP) (Cambareri et al., 1989), and RNA interference (RNAi) (Fulci and Macino, 2007) help to suppress excessive TE activity. The extensive presence of repetitive elements provides ample substrate for the RIP machinery to act upon, potentially driving the high RIP-affected genomic proportion observed in this species. *Colletotrichum lindemuthianum* has the characteristics described by van Wyk et al. (2021) for fungi that present extensive RIP: It performs sexual reproduction; has a genome rich in repetitive elements; has genes associated with RIP, DNA methylation and RIP direct heterochromatic silencing.

The sexual cycle in *C. lindemuthianum* is described as rare. This rarity would, in theory, limit the impact of the RIP silencing mechanism on its genome. However, this theoretical limitation contrasts with the significant RIP signatures presented by the *C. lindemuthianum* genome, which are the highest among the fungi evaluated. Gladyshev and Kleckner (2017) proposed a potential mechanism for generating RIP mutations that is independent of RID (C5-methyltransferase essential for the occurrence of one of the RIP pathways) activity, and thus not reliant on the occurrence of the sexual cycle in fungi. This mechanism, orchestrated by the DIM-2 protein in collaboration with the DIM-5 (histone methyltransferase) and HP1 (heterochromatin 1 protein) proteins, could be the primary factor responsible for generating RIP mutations in the *C. lindemuthianum* genome. This may explain the robust RIP signatures observed in its genome even in the absence of a frequent sexual cycle. Given that *C. lindemuthianum* exhibits the most pronounced RIP signatures among *Colletotrichum* fungi and has a rare sexual cycle, it emerges as an intriguing model for investigating the RIP generation mechanism proposed by Gladyshev and Kleckner (2017). Further investigations into the specific mechanisms governing RIP activity in this species could provide valuable insights into the evolutionary processes driving genomic diversity and adaptation in fungi.

One crucial step in comprehending the genetics of plant-pathogen interactions involves gaining access to the genomes of both entities. In this study, we present a high-quality genome of a significant pathogen in common bean cultivation, specifically, an intriguing *Colletotrichum* species. Interestingly, the expansion in the size of *C. lindemuthianum* genome did not result from an increase in the number of genes, nor in terms of families of genes that encode known virulence factors but was mainly associated with an increase in the content of repetitive elements. The data supplied in this study will bolster future investigations into this pathosystem, enabling a more in-depth understanding of the interaction between the fungus and its host. This insight will facilitate the identification of important genes within the pathogen, serving as potential targets for novel strategies aimed at disease control.

## Data Availability

The original contributions presented in the study are publicly available. This data can be found at the National Center for Biotechnology Information (NCBI) using accession number BioProject: PRJNA325493.

## Supplementary material

Supplementary Figure 1 Analysis of the repertoires of *Colletotrichum lindemuthianum* effector candidates (ClECs). (A) Comparison of the number ClECs predicted by Queiroz et al. (2019) (pink circle) and the current assembly genome (blue circle). The intersection value between the two circles represents the number of proteins sharing 100% similarity. (B) Distribution of *Colletotrichum lindemuthianum* effector candidates across different categories. The bar chart shows the total number of identified effector candidates and their distribution across various functional and subcellular categories, including secreted (secreted), apoplastic (apoplastic), cytoplasmic (cytoplasmic), nuclear (localizer_nucleus), chloroplastic (localizer_chloro), and mitochondrial (localizer_mito) effectors. “Phibase_genes” represents candidates with genes linked to the PHI-base database, while “effector_matches” indicates candidates matching known effectors.

Supplementary Figure 2 Distribution of hybrid secondary metabolite (SM) gene clusters predicted in different fungi using antiSMASH (fungal version). The numbers inside the boxes represent the number of hybrid secondary metabolite clusters found of each type, and the color intensity is related to the number. NRPS: Non-ribosomal peptide synthetases; T1PKS: Polyketide synthases type I; T3PKS: Polyketide synthases type III; R-F: fungal-RiPP (ribosomally synthesized and post-translationally modified peptide).

Suplementary Table 1 Fungal species used for analysis.

Suplementary Table 2 Alignment-Hit table of effector comparison between candidate effectors from Queiroz et al., (2019) and current candidate effectores.

Suplementary Table 3 Details of effector candidates predicted in the *Colletotrichum lindemuthianum* first genome assembly (Queiroz et al., 2019).

Suplementary Table 4 Details of effector candidates predicted in the *Colletotrichum lindemuthianum* new genome assembly.

Suplementary Table 5 Secondary metabolites clusters in *Colletotrichum lindemuthianum*.

Suplementary Table 6 RIP index value of different fungal genomes.
