# Supplementary figures and images for: What lies behind the large genome of Colletotrichum lindemuthianum
Source: Front Fungal Biol. 2024 Oct 15;5:1459229. doi: 10.3389/ffunb.2024.1459229 (PMC11518743; doi:10.3389/ffunb.2024.1459229)

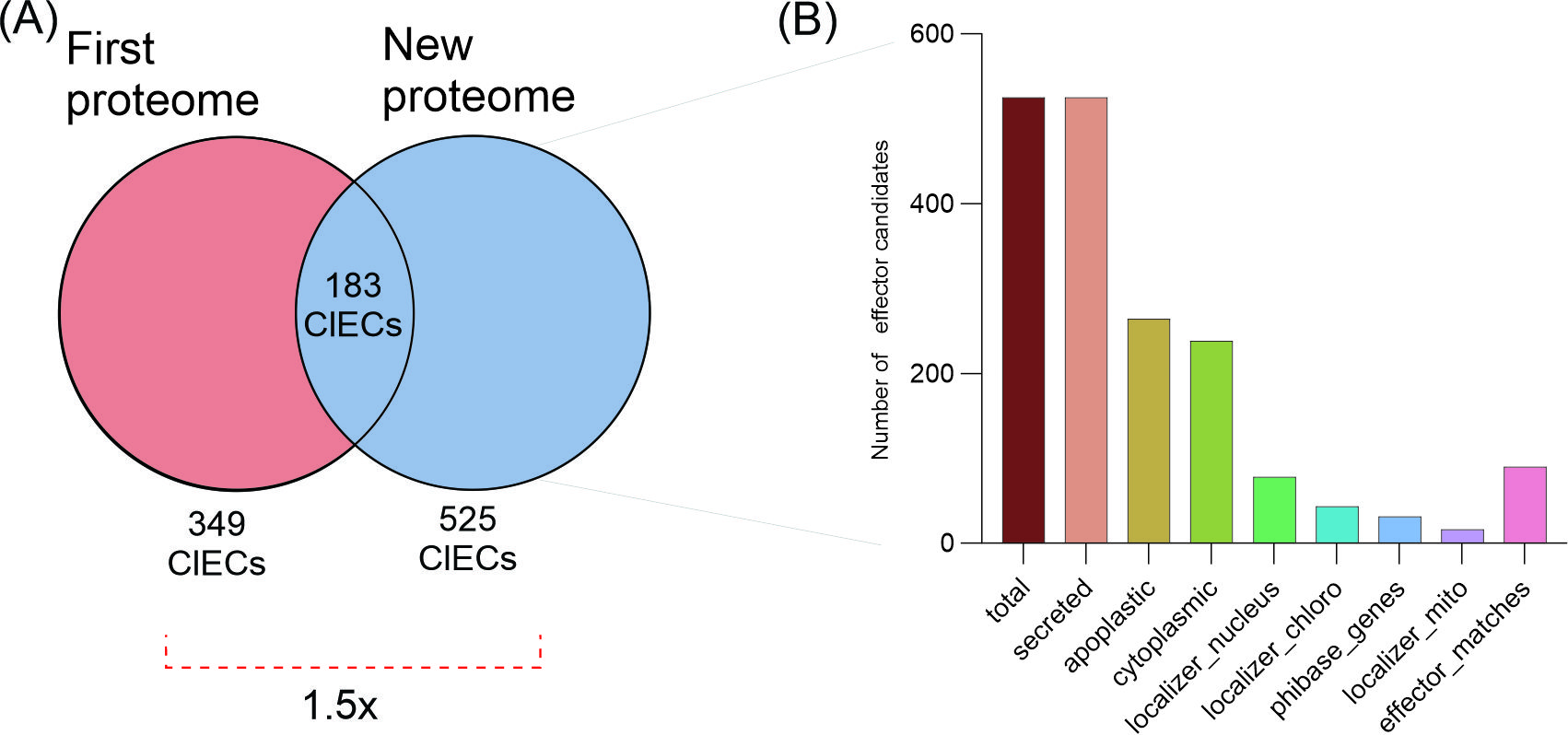

Supplement: Supplementary Figure 1 — Analysis of the repertoires of Colletotrichum lindemuthianum effector candidates (ClECs). (A) Comparison of the number ClECs predicted by Queiroz et al. (2019) (pink circle) and the current assembly genome (blue circle). The intersection value between the two circles represents the number of proteins sharing 100% similarity. (B) Distribution of Colletotrichum lindemuthianum effector candidates across different categories. The bar chart shows the total number of identified effector candidates and their distribution across various functional and subcellular categories, including secreted (secreted), apoplastic (apoplastic), cytoplasmic (cytoplasmic), nuclear (localizer_nucleus), chloroplastic (localizer_chloro), and mitochondrial (localizer_mito) effectors. “Phibase_genes” represents candidates with genes linked to the PHI-base database, while “effector_matches” indicates candidates matching known effectors. [file Image1.jpeg]

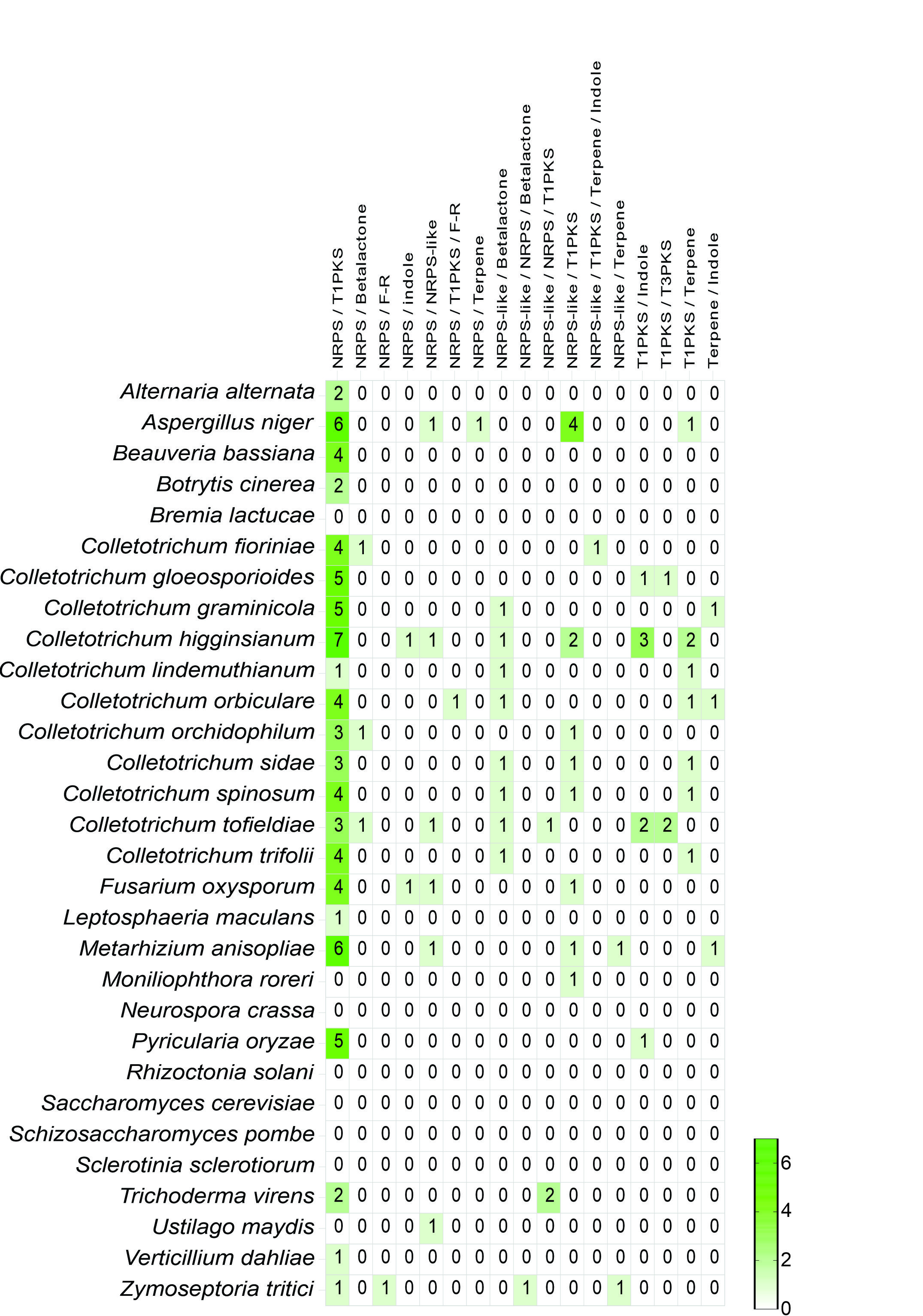

Supplement: Supplementary Figure 2 — Distribution of hybrid secondary metabolite (SM) gene clusters predicted in different fungi using antiSMASH (fungal version). The numbers inside the boxes represent the number of hybrid secondary metabolite clusters found of each type, and the color intensity is related to the number. NRPS: Non-ribosomal peptide synthetases; T1PKS: Polyketide synthases type I; T3PKS: Polyketide synthases type III; R-F: fungal-RiPP (ribosomally synthesized and post-translationally modified peptide). [file Image2.jpeg]
